# The relationship between gait task performance and AD plasma biomarkers in cognitively unimpaired older adults and patients with mild cognitive impairment

**DOI:** 10.1002/alz70856_107578

**Published:** 2026-01-09

**Authors:** Savannah Doster, Ashley N. Price, Jordan P. Sergio, Maeve Durkin, Jennifer Strenger, Louisa I. Thompson, Megan Stradtman, Stuart Sinoff, Peter J. Snyder, Jessica Alber

**Affiliations:** ^1^ University of Rhode Island, Kingston, RI, USA; ^2^ Butler Hospital Memory and Aging Program, Providence, RI, USA; ^3^ University of Rhode Island, NARRAGANSETT, RI, USA; ^4^ Alpert Medical School of Brown University, Providence, RI, USA; ^5^ Butler Hospital, Providence, RI, USA; ^6^ BayCare Health, Clearwater, FL, USA; ^7^ George & Anne Ryan Institute for Neuroscience, Kingston, RI, USA

## Abstract

**Background:**

Gait impairments in Alzheimer's disease (AD) and related dementias pose a major fall risk/contribute to morbidity/mortality. The Timed Up and Go (TUG) test is often used to assess mobility, gait changes, and dual‐task performance. The TUG‐Dual Task (TUG‐DT) version adds serial subtraction exercises to evaluate dual‐task cost (DTC). For cognitively unimpaired (CU) individuals or those with mild cognitive impairment (MCI), plasma biomarkers like pTau217, pTau181, and neurofilament light chain (NfL) can help assess the risk of AD. This study aimed to explore the relationship between TUG performance and plasma biomarkers in CU and MCI patients.

**Methods:**

Participants included CU low‐risk (*n* = 75), CU high‐risk (*n* = 87), and CI (*n* = 32) older adults aged 55‐80, mean = .67.28 ± 6.062 years. Cognitive ability was assessed using the Clinical Dementia Rating Scale (CU = 0; CI = 0.5 or 1.0) and the Montreal Cognitive Assessment (CU ≥ 26; 18 ≤ CI ≤ 26). AD‐risk was determined by APOE genotyping and family history for CU groups. Plasma biomarkers pTau217, pTau181, and NfL were analyzed from fasting blood draws. Participants completed the TUG and TUG‐DT. ANOVAs, ANCOVAs, logistic regression, and generalized additive models (GAMs), were used to analyze the relationship between demographic factors, gait performance, and plasma biomarkers, with model comparisons guiding the final choice of GAMs for their flexibility in handling non‐linear relationships.

**Results:**

Step count analysis on the TUG showed that the CU‐high‐risk and MCI groups performed similarly, while the CU‐low‐risk group completed significantly fewer steps than both. Plasma biomarkers, particularly pTau181 and NfL, interacted to predict gait performance only in the CU high‐risk group.

**Conclusions:**

The TUG can predict plasma pTau217 levels with high specificity, distinguishing CU individuals not at risk for AD. Additionally, pTau181 and NfL interacted to predict performance on the TUG and TUG‐DT in the CU‐high‐risk group, suggesting subtle gait changes may signal early AD pathology. The CU‐low‐risk group's reduced step count compared to others indicates preclinical AD might manifest with subtle mobility impairments. These findings support using simple gait tasks like the TUG for AD risk assessment in older adults.

